# Integrating education and conservation: a case study of the Huaper wetland

**DOI:** 10.3389/fpsyg.2025.1517653

**Published:** 2025-03-06

**Authors:** Bruno Kadafi Cardenas Morales, Saríah Fanny Oré Gálvez, Walter Victor Castro Aponte, Antony Aguilar Ozejo, Rubén Ñaupari Molina, Fernando Gari Huayhua Lévano, Manuel Mendoza Colos

**Affiliations:** Facultad de Ingeniería y Gestión, Escuela Profesional de Ingeniería y Gestión Ambiental, Universidad Nacional Autónoma de Huanta, Huanta, Peru

**Keywords:** environmental education, wetland conservation, ecosystem services, learning methodologies, community engagement

## Abstract

Wetlands provide essential ecosystem services such as biodiversity conservation, water regulation, and agricultural support. However, in Peru, wetland degradation due to urbanization and unsustainable practices threatens these vital functions. This study applies an experiential environmental education approach to wetland conservation, using the Huaper Wetland as a case study. The research is grounded in constructivist learning theories and evaluates the wetland’s biophysical condition while implementing hands-on educational activities to promote student engagement and community participation. Findings indicate that immersive learning experiences enhance ecological understanding and encourage sustainable behaviors. Additionally, the study identifies key factors influencing the effectiveness of educational interventions in wetland conservation. The research proposes four actionable conservation strategies that align educational efforts with community priorities. The study highlights experiential education as both a conservation tool and a means of transformative learning, offering a replicable model for sustainable wetland management.

## 1 Introduction

Environmental education (EE) emerges as a vital strategy to address the pressing global environmental crisis. As noted by Adhya and Banerjee (2020), EE not only enhances understanding of environmental impacts but also fosters sustainable practices by creating emotional and ethical connections with nature. Grounded in constructivist principles, EE integrates theoretical knowledge with experiential learning, as evidenced by Mandishona and Knight (2019) and Saluja et al. (2023). This approach enables individuals and communities to develop long-term sustainable behaviors, particularly in the context of ecosystems under threat.

Within the framework of constructivist learning theories, experiential education emphasizes the importance of direct interaction with nature to foster deep, meaningful learning (Rode, 2020; Gabites and Spencer, 2020). This pedagogical approach aligns with psychological frameworks, emphasizing active participation and collaboration to enhance engagement and retention of knowledge (Díaz-Pinzón et al., 2022). Wetlands, with their unique ecological characteristics, provide an ideal setting for this form of education, serving as both natural classrooms and critical ecosystems in need of conservation (Balwan and Kour, 2021).

Wetlands are essential for biodiversity, climate regulation, and the provision of vital ecosystem services, such as water filtration, flood protection, and carbon sequestration (De et al., 2018).

Despite their significance, urbanization, pollution, and climate change have accelerated their degradation worldwide (Adhya and Banerjee, 2020; Gao et al., 2023). As highlighted by Adeeyo et al. (2022), the loss of wetland functionality threatens both ecological integrity and human livelihoods, necessitating urgent, evidence-based conservation strategies.

The Huaper wetland, located in the community of Azángaro, Luricocha district, Huanta province, exemplifies the challenges faced by wetlands at the regional level. Issues such as solid waste accumulation, declining water levels, intensive grazing, and limited environmental awareness among local residents mirror global trends (Dadzie-Paintsil and Mensah, 2022; Mandishona and Knight, 2019). These challenges underscore the need for targeted conservation initiatives that combine community engagement with robust environmental education programs.

This study aims to bridge the gap between ecological understanding and practical conservation by integrating experiential environmental education into wetland management. By assessing the Huaper wetland’s ecological conditions and engaging students in immersive learning activities, this research seeks to demonstrate the potential of constructivist education to foster sustainable behaviors. Through collaboration with the local community and educational institutions, the findings contribute to the broader discourse on wetland conservation, offering a replicable model for integrating education and ecological stewardship.

## 2 Materials and methods

### 2.1 Study area

**Figure 1:**
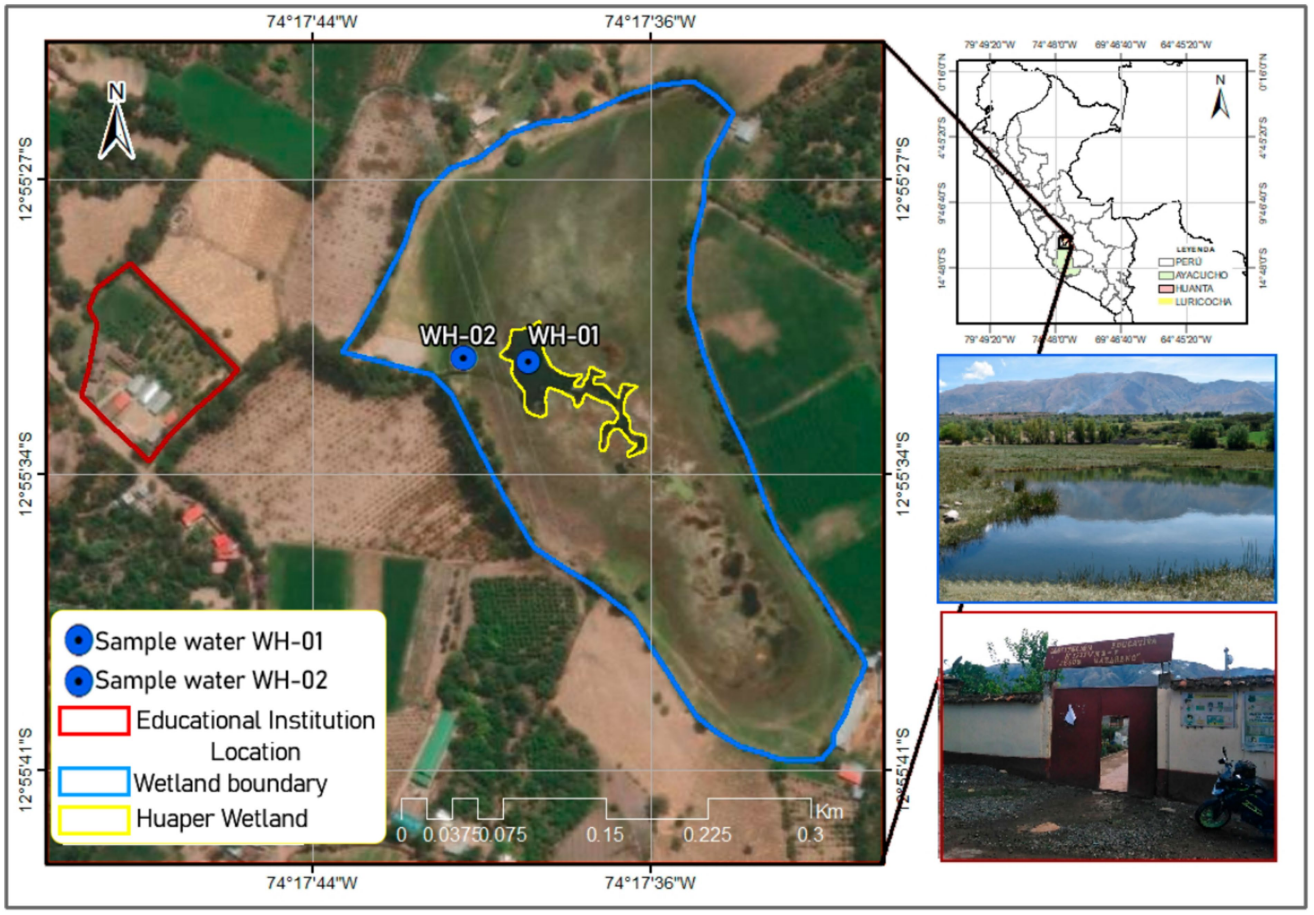
Location of the Huaper Wetland, sampling points, and the ‘Corazón de Jesús’ educational institution.

The Huaper wetland is located in the community of Azángaro, Luricocha district, Huanta province, Ayacucho region, Peru. This ecosystem lies at an altitude of 2,353 meters above sea level, covering an approximate area of 84,197 m^2^, and is part of the hydrological system of the Cachi and Mantaro river basins (Shrestha, 2013). The wetland consists of a large central water body and a smaller lagoon (WH-01 and WH-02, respectively), both primarily fed by groundwater from the upper regions of Huanta (Figure 1). The groundwater accumulates through precipitation infiltration, which percolates through the soil until it reaches saturated rock layers. This groundwater flow emerges in the wetland through natural springs and drainage points.

The ecosystem belongs to the Iribamba plain and plays a key role in regulating the hydrological cycle, maintaining biodiversity, and supporting human activities such as agriculture and livestock. Geomorphologically, the area features steep mountain slopes, colluvial deposits, and alluvial plains. These formations have been shaped by both endogenous and exogenous processes, including fluvial erosion and human activity (Ramírez and Santana, 2019).

The wetland hosts a rich biodiversity, including 20 species of vascular flora, such as totora (*Schoenoplectus californicus*), water hyacinth (*Eichhornia crassipes*), rush (*Juncus effusus*), aquatic fern (*Salvinia minima*), and duckweed (*Lemna minor*). These floating and emergent species contribute to the ecological stability of the wetland and provide vital aquatic habitats.

The fauna of the wetland is equally diverse, featuring species such as the great egret (*Ardea alba*), the white-faced ibis (*Plegadis chihi*), the common gallinule (*Gallinula chloropus*), and the striped cuckoo (*Tapera naevia*). These birds find refuge and food in the swampy meadows and totora reed beds, which are essential components of the wetland ecosystem.

The wetland also holds significant cultural and educational importance for the local community due to its proximity (320 meters) to the “Jesús Nazareno” educational institution. This close distance has facilitated its use as an open-air classroom for experiential learning activities that combine theory and practice, fostering environmental education and conservation efforts (Shrestha, 2013).

### 2.2 Water analysis

The assessment of water quality in the Huaper wetland integrated direct observations and laboratory analyses to provide a comprehensive understanding of the ecosystem’s current state. Water samples were collected from two representative sites: the central water body (WH-01), representing the core of the ecosystem, and a secondary lagoon (WH-02), reflecting areas potentially more vulnerable to anthropogenic impacts. These sites were selected based on prior reconnaissance to ensure adequate representation of spatial variability. Sampling was conducted in May 2023 during the early morning hours (7,00–9,00 AM) to minimize diurnal variations in water quality parameters, following established protocols (McIntyre, 2004).

Sterile 500 mL glass bottles were used for sample collection, following aseptic protocols to prevent contamination. Each site was sampled once, with three replicates taken per site to enhance the reliability and precision of the measurements. Key water quality parameters analyzed included pH, dissolved oxygen (DO) expressed as both percentage and ppm, and electrical conductivity (EC) measured in μS/cm. All measurements were performed using a Hanna HI98194 multiparameter probe, which was calibrated before sampling using standard buffer solutions (pH 4.01 and 7.00) and conductivity standards. Calibration and verification of the device were conducted against laboratory-grade equipment at the National Autonomous University of Huanta to ensure accuracy.

**Figure 2:**
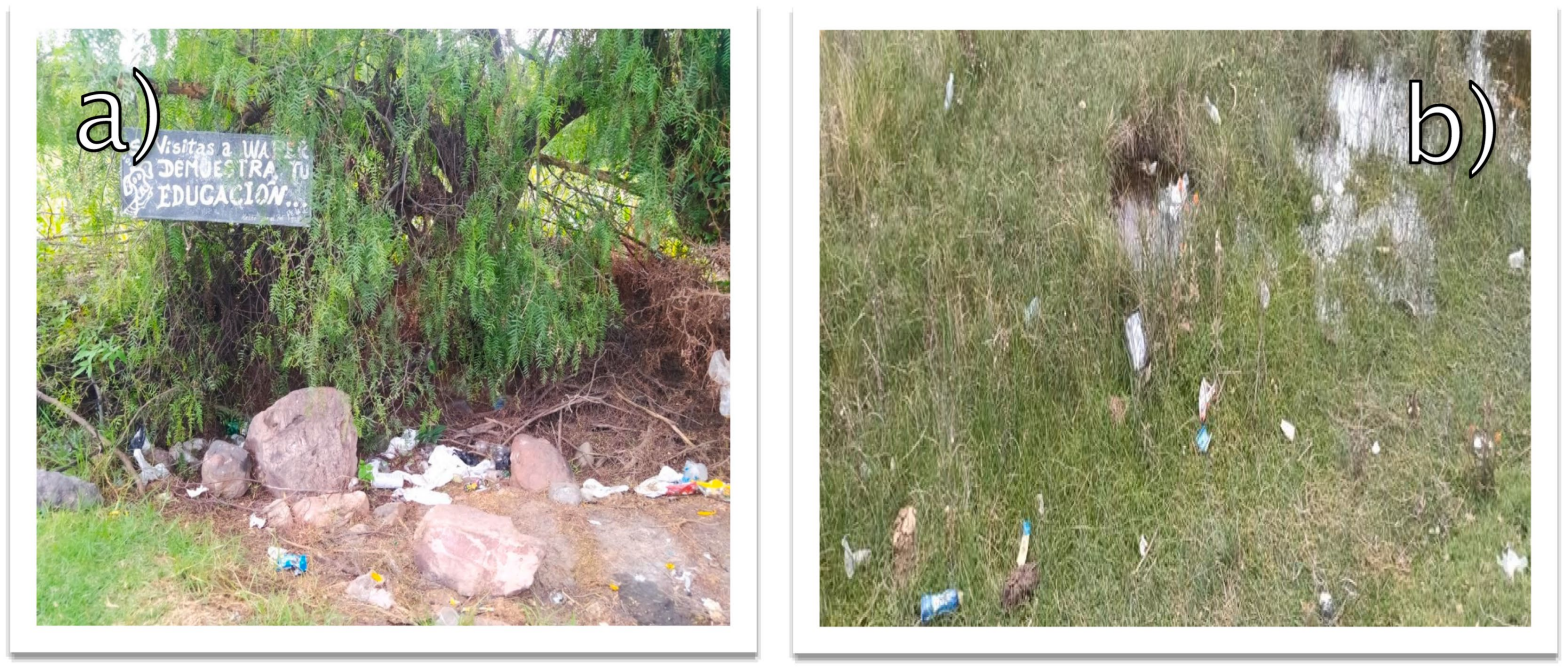
**(A)** Accumulation of ex-situ solid waste near the Huaper wetland. **(B)** Visible in-situ solid waste embedded in wetland vegetation.

In addition to water sampling, direct observations documented visible signs of pollution, including solid waste accumulation near access points and within the wetland (Figure 2). These observations were guided by standardized field protocols to ensure consistent data collection (McIntyre, 2004). Photographic evidence highlights these issues, as seen in Figure 2, which shows ex-situ solid waste such as plastics and organic debris near visitor areas (Figure 2A), as well as in-situ waste embedded in vegetation and waterlogged zones (Figure 2B), illustrating the wetland’s vulnerability to contamination. Observations were conducted during daylight hours to maximize natural lighting and ensure precise documentation.

This study did not account for seasonal variations, as sampling was limited to a single season. Future research should incorporate longitudinal sampling across different seasons to capture temporal dynamics in water quality. Despite this limitation, the combination of observational and analytical methods provides a valuable baseline for understanding the ecological challenges facing the Huaper wetland and informs targeted conservation strategies.

### 2.3 Integration of water analysis and educational activities

Water quality analysis with environmental education activities was a central component of the project. Direct observations conducted in the Huaper wetland, particularly the identification of visible solid waste and potential pollution sources, informed the initial classroom discussions. These discussions provided students with a contextual understanding of the environmental issues affecting the wetland.

The results of the water quality analysis, including parameters such as pH, dissolved oxygen, and electrical conductivity, were presented to the students in subsequent sessions. This data was used to facilitate interactive discussions on the impact of water quality on wetland ecosystems and the importance of maintaining clean water resources. Students were encouraged to interpret the data, identify potential environmental risks, and propose solutions. By linking scientific data with experiential learning, the program fostered critical thinking and a deeper appreciation of the wetland’s ecological value.

### 2.4 Collaboration with the educational institution “Jesús Nazareno”

The collaboration with the Educational Institution “Jesús Nazareno” was a cornerstone of this study, as the school’s proximity to the Huaper wetland (approximately 320 meters away) enabled seamless integration of theoretical and practical learning activities. This public primary school, managed directly by the Ministry of Education, serves students from the rural community of Azángaro, in the district of Luricocha, province of Huanta, Ayacucho. The school operates under challenging socioeconomic conditions, with limited resources and infrastructure, reflecting the broader economic difficulties faced by the region.

The institution serves both male and female students and operates exclusively during morning hours. Most students come from low-income families engaged in subsistence agriculture and livestock activities, which underscores the importance of integrating environmental education into their curriculum. Despite these challenges, the school provided a supportive environment for the implementation of experiential learning activities, enabling students to connect their classroom knowledge with real-world environmental issues.

Teachers played a pivotal role in the project, acting as facilitators during both classroom sessions and field activities. Their involvement ensured that the educational content was adapted to the students’ cognitive development levels and aligned with the national curriculum for environmental education. The community also showed active participation, with parents and local leaders expressing interest in the project and contributing to the broader dissemination of environmental conservation practices.

Two student groups were organized for this study: one consisting of 15 students from 5th and 6th grades and another with 15 students from 3rd and 4th grades. These groups participated in activities tailored to their cognitive development stages, allowing for an age-appropriate understanding of environmental conservation concepts (Khoshkam and Marzuki, 2011). The structure of these groups facilitated collaborative learning and fostered a sense of shared responsibility among students for the conservation of the Huaper wetland.

### 2.5 Survey of students at the educational institution “Jesús Nazareno”

To assess the level of environmental knowledge and perception of the students regarding the Huaper wetland, a structured survey was carefully designed and applied. The survey focused on exploring students’ prior knowledge about specific aspects of the wetland, such as its ecosystem services, its role in the community’s well-being, and basic principles of conservation. This baseline information was essential for tailoring subsequent educational activities to the students’ needs and comprehension levels.

**Figure 3:**
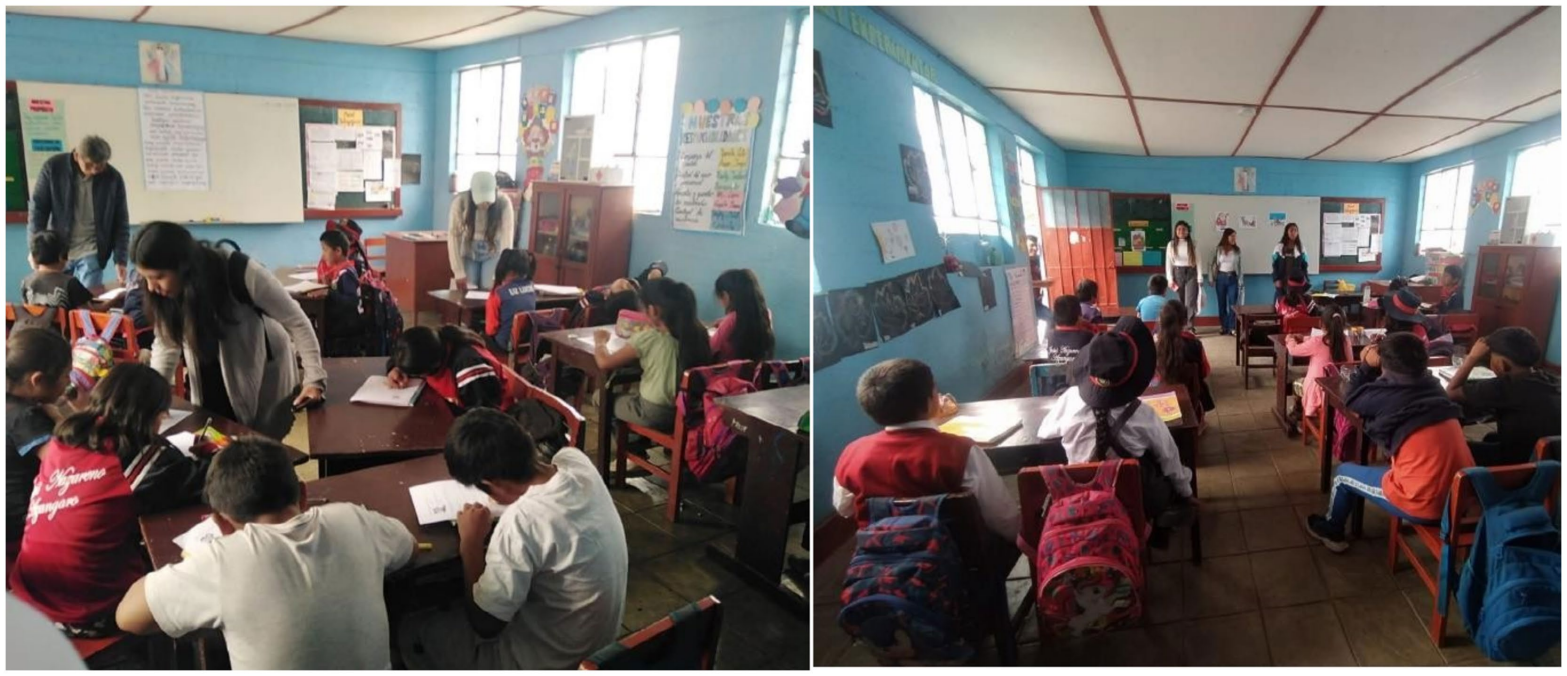
Students completing the survey in the classroom under the supervision of both the research team and teachers.

The survey was conducted in the classroom of the Educational Institution “Jesús Nazareno,” under the supervision of the research team and teachers (Figure 3). A total of 30 students participated, divided into two groups of 15 students each: one group comprised students from 5th and 6th grades, and the other from 3rd and 4th grades. Each figure in the results section represents data for a single group, with *n* = 15 students per group. This grouping reflects the school’s structure, which combines four grades into two main clusters due to its small size. This structure also allowed for the adaptation of educational activities to different cognitive levels while maximizing the diversity of insights gathered.

#### 2.5.1 Clarification regarding data analysis

The survey data were analyzed separately for each group (*n* = 15), consisting of students from 3rd and 4th grades in one group and 5th and 6th grades in another. Figures in the results section reflect these distinctions, presenting pre- and post-education program comparisons for each group independently. This approach ensures that the analysis accurately represents the specific learning outcomes within each cluster, aligning with the study’s objective to tailor educational interventions to different cognitive levels.

Each student completed the survey in approximately 15 min, ensuring they had ample time to understand and answer the questions properly. The survey included both multiple-choice and open-ended questions. Multiple-choice questions assessed factual knowledge about topics such as biodiversity, water quality, and environmental challenges, while open-ended questions explored students’ perceptions and attitudes toward the conservation of the Huaper wetland. This mixed-methods approach allowed for a comprehensive understanding of the students’ perspectives and the identification of key areas for improvement.

The findings of this survey provided crucial insights that guided the customization of educational workshops and field activities. Addressing specific knowledge gaps and misconceptions ensured that the activities were relevant and impactful for the students’ learning process.

### 2.6 Analysis of survey data

The survey data were analyzed using both quantitative and qualitative methods to gain a comprehensive understanding of the students’ environmental knowledge and attitudes.

#### 2.6.1 Quantitative analysis

Quantitative data derived from multiple-choice questions were analyzed using Microsoft Excel, a software chosen for its accessibility and capability to calculate descriptive statistics such as frequencies, percentages, and mean values. These metrics provided insights into students’ factual understanding of key topics, including biodiversity, water quality, and environmental challenges.

The analysis was performed separately for each group (*n* = 15), representing students from 3rd and 4th grades in one group and 5th and 6th grades in another. Additionally, pre- and post-education program comparisons were conducted to assess knowledge acquisition over time, ensuring clarity and consistency in data interpretation.

Since the primary objective was to describe general patterns rather than establish statistical significance, no inferential statistical tests were performed due to the limited sample size.

#### 2.6.2 Qualitative analysis

For the open-ended questions, a manual thematic coding approach was employed to ensure an in-depth analysis of students’ perceptions. Responses were iteratively reviewed by the research team to minimize bias and identify recurring themes and patterns. Key themes included:

- Perceptions of the ecological importance of the wetland.
- Attitudes toward conservation efforts.
- Suggestions for addressing environmental challenges.

For instance, comments such as “The wetland provides water for animals and plants” were categorized under ecosystem services, while concerns about pollution were grouped under conservation challenges.

#### 2.6.3 Triangulation and validation of findings

The results from the quantitative analysis were cross-referenced with the qualitative findings, observational data from educational activities, and direct feedback from students and teachers. This process validated the consistency of the knowledge and attitudes reported by students across different data sources.

Additionally, iterative review sessions within the research team were conducted to enhance the accuracy of thematic coding and ensure that the identified themes reflected the underlying data comprehensively.

##### 2.6.3.1 Limitations

While the manual coding process allowed for nuanced insights, it is subject to potential researcher bias. Furthermore, the small sample size limited the generalizability of the findings beyond the studied context. Future studies could incorporate software tools like NVivo for qualitative analysis to ensure more systematic coding and reduce potential biases.

The study utilized pre and post surveys to evaluate the knowledge levels of students before and after the implementation of the educational program. However, the absence of a control group—students in the same academic stage who did not participate in the program—limits the ability to isolate the program’s impact from other external factors. This limitation was primarily due to the small population of students available in the study area, making it challenging to assign separate groups without compromising the statistical validity of the findings.

Despite this limitation, the use of pre- and post-surveys provided valuable insights into the program’s effectiveness by directly comparing changes in knowledge levels within the same cohort. Triangulation with observational data and feedback from students and teachers further strengthened the validity of the findings. Future research should aim to address these limitations by incorporating control groups and larger sample sizes, enabling a more comprehensive evaluation of the program’s effectiveness and generalizability.

#### 2.6.4 Educational workshops and field activities

The educational program incorporated a series of structured activities that seamlessly integrated theoretical classroom sessions with practical fieldwork at the Huaper wetland. These activities were conducted over four separate visits to the wetland, spanning approximately 1 month, ensuring a progressive and comprehensive learning experience. The program was divided into three distinct phases preparation, execution, and reflection to align with the study’s educational goals and provide students with a meaningful connection to their local environment.

##### 2.6.4.1 Phase 1: classroom preparation

The first phase involved two classroom sessions where students were introduced to essential concepts of wetland ecology, including biodiversity, hydrological cycles, and ecosystem services. These lessons used visual aids, such as maps and photographs of the Huaper wetland, alongside discussions of real-world challenges like pollution and declining biodiversity. Students also received an overview of the field activities, ensuring they understood the objectives and methodologies of their wetland visits. This phase set a strong conceptual foundation for their practical experiences (Lu and Wang, 2015).

##### 2.6.4.2 Phase 2: field activities

Fieldwork was conducted over four visits to the Huaper wetland, each lasting approximately 4 h. These visits were spaced over 4 weeks to allow time for reflection and integration of the findings between sessions. The activities conducted during the visits included:

1. **First Visit: Environmental Observation and Mapping**
  Students were tasked with observing the wetland’s overall state, documenting visible pollution sources, and mapping key features such as vegetation zones and water bodies. This visit provided an introductory exploration of the wetland and set the stage for subsequent activities.
2. **Second Visit: Water Quality Analysis**
  During this visit, students collected water samples from two representative sites within the wetland and measured parameters such as pH, electrical conductivity, and dissolved oxygen. The data collection followed standardized protocols and was supervised by the research team and teachers.
3. **Third Visit: Flora and Fauna Documentation**
  Students conducted detailed observations of the wetland’s flora and fauna, using photographs and written records to document their findings. This activity aimed to deepen their understanding of biodiversity and the ecological importance of the wetland.
4. **Fourth Visit: Problem-Solving and Conservation Planning**
  The final visit focused on synthesizing the data and observations collected during the previous visits. Students engaged in group discussions to identify the wetland’s primary environmental challenges and propose actionable conservation strategies.

##### 2.6.4.3 Phase 3: reflection and synthesis

In the weeks following the field visits, students participated in three classroom workshops to consolidate their findings. Activities included:

- **Data Analysis and Interpretation:** Students analyzed the water quality data and compared their observations of flora and fauna to identify trends and patterns.
- **Creative Projects:** Students created educational posters and presentations highlighting the wetland’s importance and the challenges it faces.
- **Collaborative Discussions:** Guided discussions allowed students to share insights and reflect on how their learning experience could translate into actionable conservation efforts.

By structuring the program into these phases and organizing multiple visits, the educational initiative was designed to progressively engage students with the Huaper wetland. This approach aimed to ensure that students could connect theoretical knowledge with practical experiences in a structured and collaborative manner.

#### 2.6.5 Workshops for applying and synthesizing knowledge

Following the field activities, workshops were organized to help students consolidate the knowledge gained during their exploration of the Huaper wetland. In these workshops, students engaged in creating wetland models and educational posters illustrating the ecosystem services provided by the wetland. These activities served as platforms for collaborative work, fostering creativity and reinforcing the concepts discussed during the classroom and field sessions.

The workshops were strategically held at the end of the educational program, allowing students to connect their theoretical understanding with practical observations from the field. This iterative process reflects the principles of project-based and experiential learning, enabling students to internalize their knowledge in a dynamic and tangible way (Wightman et al., 2015).

By constructing models and posters, students not only visualized complex ecological concepts but also enhanced their ability to communicate environmental issues effectively. Feedback from students indicated that these activities deepened their engagement and understanding, aligning with the goals of fostering environmental awareness and stewardship.

### 2.7 Interviews with visitors to the Huaper wetland

Semi-structured interviews were conducted with a purposive sample of 22 individuals, including recreational visitors and local residents, to gain qualitative insights into environmental perceptions, waste management practices, and attitudes toward conservation. This method allowed participants to share their experiences and opinions in detail, capturing nuances that quantitative surveys could not provide.

**Figure 4:**
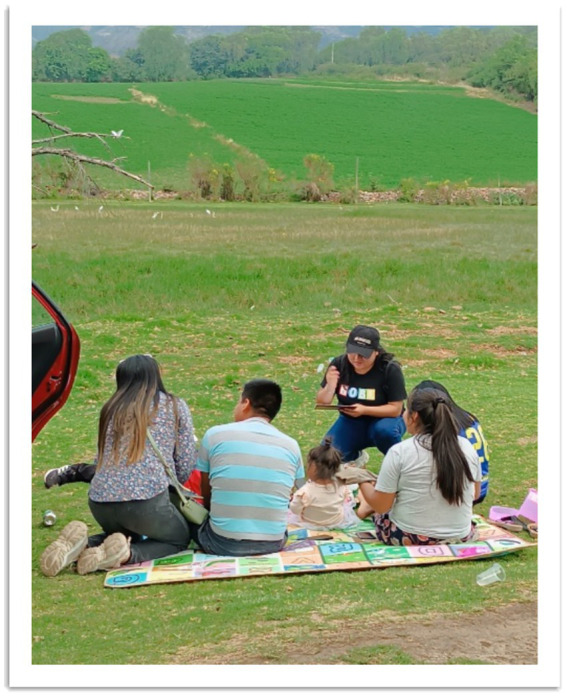
Interviews with visitors to the Huaper Wetland, illustrating the diverse group of participants and the setting for qualitative data collection.

The interviews, lasting 15 to 20 min each, addressed key themes such as waste disposal practices, biodiversity awareness, and the frequency and purpose of visits to the wetland. This qualitative approach enriched the study by complementing observational data and student surveys, providing a more holistic understanding of the wetland’s socio-environmental dynamics. Additionally, it contextualized the educational outcomes of the project, aligning with methodologies in similar studies of urban wetlands (Mandishona and Knight, 2019; Sinthumule, 2022). The qualitative methodology allowed for capturing nuanced insights into visitors’ environmental perceptions and interactions with the wetland. These interviews contextualized the obtained results and complemented observational data and student surveys (see Figure 4).

#### 2.7.1 Ethical considerations

Consent for participation was obtained through a detailed briefing process, ensuring that all participants or their guardians understood the study’s objectives, methods, and their rights. Special care was taken to adapt consent forms for minors, with teachers assisting in the process to ensure clear communication. These measures adhered to ethical guidelines set by the National Autonomous University of Huanta.

#### 2.7.2 Qualitative data analysis

The interview data were analyzed using a thematic coding approach to identify recurring patterns and insights. Key themes included concerns about inadequate infrastructure for waste management, the perceived need for enhanced environmental education, and varying levels of awareness regarding the ecological importance of the Huaper wetland. These findings are consistent with prior studies emphasizing how socioeconomic and educational factors influence community knowledge and conservation attitudes (Truong, 2021).

The coding process was conducted iteratively to ensure accuracy and consistency. Themes were triangulated with data from student surveys and observational findings to enhance reliability. This triangulation provided a comprehensive understanding of community interactions with the wetland and reinforced the study’s conclusions.

### 2.8 Theoretical framework and methodological application

#### 2.8.1 Constructivist principles in methodology

The constructivist theory of learning, proposed by theorists such as Piaget and Vygotsky, emphasizes that knowledge is actively constructed by learners through their interactions with the environment. This study’s methodology applied constructivist principles to design educational activities that engaged students in hands-on, reflective learning experiences. Activities such as water quality analysis, biodiversity documentation, and pollution mapping were central to the project, enabling students to connect theoretical knowledge with real-world challenges.

The project’s focus on wetland conservation provided a dynamic platform for constructivist learning. Through active engagement with the wetland ecosystem, students explored complex environmental concepts, such as the interdependence of biodiversity and water quality. This methodological alignment ensured that the activities fostered critical thinking and meaningful connections to environmental stewardship.

#### 2.8.2 Cognitive development and Piaget’s framework

Jean Piaget’s theory of cognitive development served as a foundation for tailoring activities to the cognitive abilities of students in different age groups. The methodology divided activities based on Piaget’s developmental stages to ensure that tasks were age-appropriate and cognitively engaging:

- **Preoperational Stage (3rd and 4th Grades):** Younger students participated in symbolic and manipulative exercises, such as pollution clean-up simulations and classification of wetland elements using tangible materials. These activities facilitated direct interaction with natural elements, helping students to internalize basic ecological concepts (Santana and Obara, 2017).
- **Concrete and Formal Operational Stages (5th and 6th Grades):** Older students conducted water quality analysis, measured parameters such as pH and dissolved oxygen, and documented biodiversity using observational tools. These activities required higher-order thinking skills, including data interpretation and problem-solving, reflecting the cognitive demands of Piaget’s concrete and formal operational stages (Velázquez Labrada, 2016).

This staged approach ensured a progression in cognitive complexity and aligned with the students’ developmental capacities, fostering deeper ecological understanding.

#### 2.8.3 Experiential learning and John Dewey’s philosophy

The field activities were designed in accordance with John Dewey’s philosophy of experiential learning, which emphasizes inquiry-based and real-world problem-solving education. By grounding the methodology in Dewey’s principles, the project sought to empower students to actively engage with their environment and develop practical solutions to wetland conservation issues.

**Figure 5:**
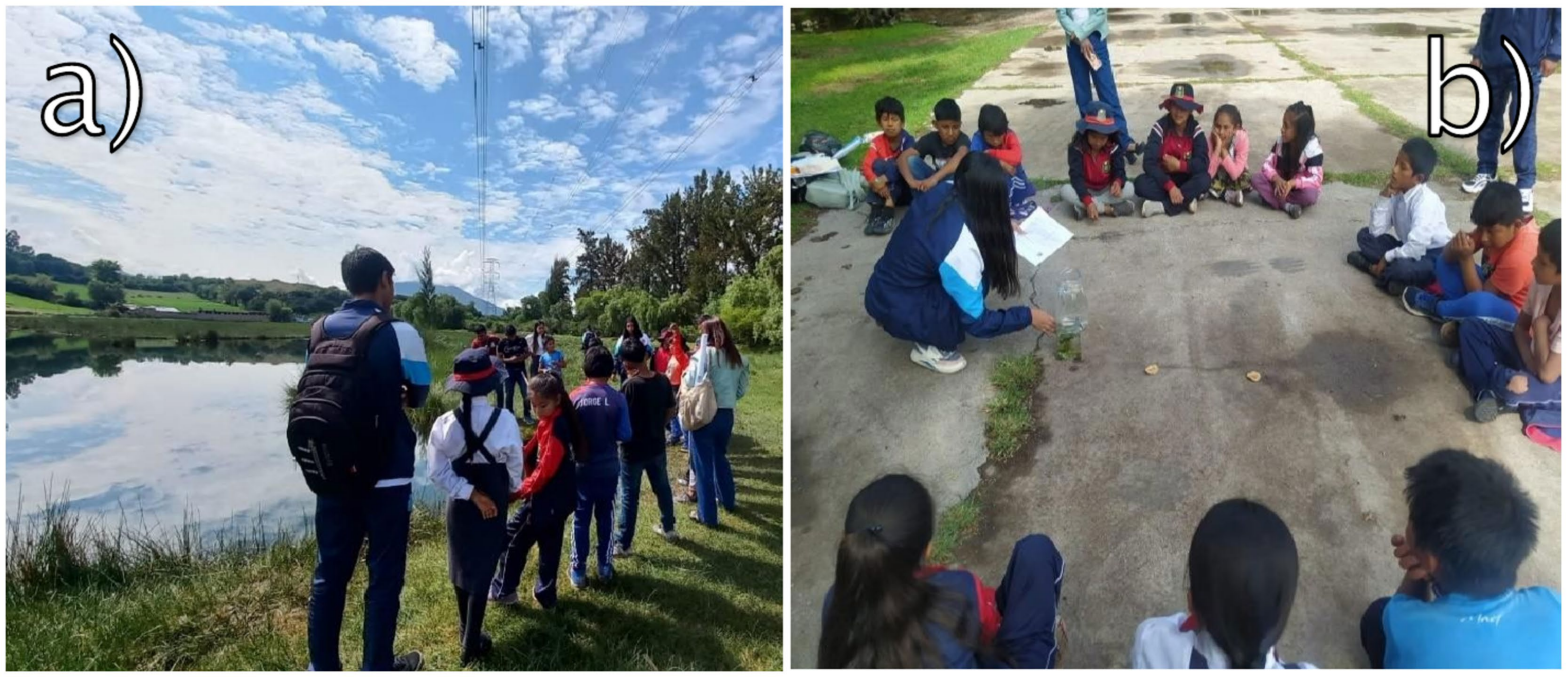
Experiential sessions with students. **(A)** Representing Piaget’s framework, younger students engaged in symbolic and manipulative activities, such as observing and classifying wetland elements. These activities facilitated the development of cognitive skills and an early appreciation of ecological systems. **(B)** Reflecting Dewey’s principles, older students collaborated in group discussions to analyze data and propose conservation strategies. This collaborative and reflective process fostered critical thinking and real-world problem-solving.

Field sessions included collecting water samples, documenting plant and animal species, and analyzing pollution sources. For instance, students hypothesized about the causes of water quality variations and tested their hypotheses through field measurements. This iterative process of data collection, reflection, and group discussion reinforced a sense of responsibility and agency in addressing environmental challenges (Young and Malone, 2023) (Figure 5).

#### 2.8.4 Interdisciplinary approach and community collaboration

To enhance the educational methodology, the project adopted an interdisciplinary framework integrating biology, geography, and social sciences. This approach enabled students to understand the interplay between natural systems and human activities, fostering a holistic perspective on wetland conservation.

Community collaboration was a key component of the methodology. Parents, educators, and local experts participated in workshops and field sessions, sharing insights and experiences. This collective engagement not only enriched the students’ learning process but also strengthened the community’s commitment to wetland conservation (Santana and Obara, 2017).

Additionally, the interdisciplinary approach facilitated the exchange of diverse perspectives, promoting ecosystem conservation efforts at a broader scale (Velázquez Labrada, 2016).

## 3 Results

This section presents findings in two interconnected areas: (1) the physicochemical analysis of the Huaper wetland and (2) the outcomes of the environmental education program. These results provide critical insights into the wetland’s ecological status and the effectiveness of educational activities aimed at enhancing students’ knowledge and conservation awareness.

### 3.1 Overview of findings

The physicochemical analysis highlights critical environmental conditions within the Huaper wetland, such as low dissolved oxygen levels and high ion concentrations, which suggest ecological stress. Simultaneously, the surveys reveal that students exhibit intermediate levels of knowledge about wetland conservation but show gaps in advanced understanding, particularly regarding biodiversity and pollution impacts. Together, these findings underscore the need for targeted conservation measures and enhanced educational strategies.

### 3.2 Physicochemical analysis of the Huaper wetland water

Water samples were collected from two representative sites within the Huaper wetland, and their physicochemical parameters were analyzed (Table 1). The results offer critical insights into the ecological health of the wetland and potential risks to biodiversity.

**Table 1:** Physicochemical parameters of the water from the Huaper Wetland (WH-01).

Parameters	Sample 1	Sample 2
Ph	6.93	6.93
mV ORP	291.7	271.3
%DO	2.3	2.3
ppm DO	0.13	0.20
μS/cm	866	865
T °C	20°	19°

### 3.3 Key observations and implications

The pH values of 6.93 for both samples indicate neutral conditions, typically conducive to many forms of aquatic life. However, without site-specific comparisons within the same watershed, the implications of this stability for local biodiversity remain uncertain. Research highlights the importance of regional factors, such as hydrology and vegetation, in shaping wetland pH levels (Junk et al., 2011).

The dissolved oxygen (DO) levels of 0.13 ppm and 0.20 ppm indicate significant ecological stress, likely resulting from organic matter decomposition that consumes oxygen, limiting the survival of aerobic organisms. These findings are consistent with observations in nutrient-rich wetlands experiencing high organic loads (Bhuiyan et al., 2011).

The oxidation–reduction potential (ORP) values (291.7 mV and 271.3 mV) suggest oxidative conditions that facilitate nutrient cycling. However, the disparity between these values and the critically low DO levels suggests an imbalance between decomposition processes and oxygen replenishment.

The conductivity values (866 μS/cm and 865 μS/cm) reflect elevated ion concentrations, potentially stemming from agricultural runoff and organic matter. While typical of nutrient-rich wetlands, prolonged high conductivity can favor tolerant species over sensitive ones, potentially destabilizing the ecosystem (Kentula et al., 2020).

#### 3.3.1 Implications for biodiversity and conservation

The results highlight the dual role of the Huaper wetland as a nutrient sink and a biodiversity hotspot under stress. Low DO levels pose significant risks to aquatic organisms, particularly those requiring aerobic conditions for survival. Similarly, high ion concentrations could alter community dynamics if sustained over time. These findings emphasize the necessity of regional monitoring and localized conservation efforts, aligning with recommendations from Junk et al. (2011) for wetland management in complex ecological contexts.

### 3.4 Environmental education outcomes

The educational program aimed to enhance students’ understanding of wetland conservation by integrating classroom instruction with hands-on activities. Knowledge assessments conducted among students from 3rd to 6th grades provided insights into their comprehension of key ecological topics. The results of these assessments are detailed in the corresponding sections.

#### 3.4.1 Knowledge levels of 3rd and 4th grade students (pre-education program)

**Figure 6:**
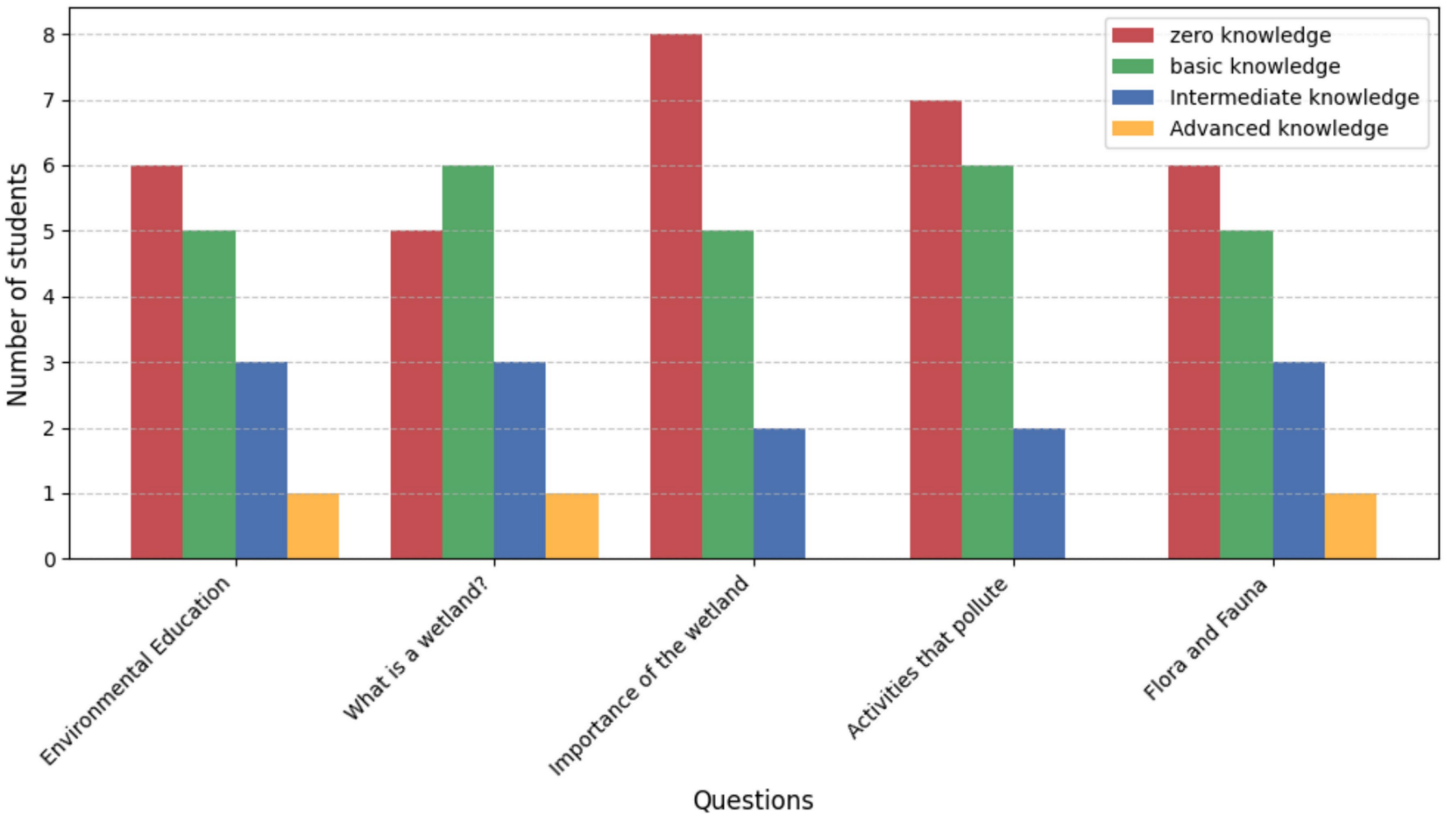
Knowledge levels of 3rd and 4th-grade students (*n* = 15) prior to the educational program.

Younger students exhibited a significant knowledge gap prior to the educational program. Among the 15 students in the 3rd and 4th-grade group (*n* = 15), zero knowledge was notable in key areas such as “What is a wetland?” and “Activities that pollute the wetland,” reflecting a lack of foundational understanding. Basic knowledge was the most common level observed, particularly in general topics like “Environmental education” and “Flora and fauna of the wetland.” However, intermediate knowledge was present in a smaller group of students, highlighting a limited ability to connect concepts like “The importance of the Huaper wetland” to practical conservation efforts. Only a single student demonstrated advanced knowledge, indicating a critical need for intervention to improve ecological awareness and understanding (Figure 6).

These findings suggest that students in the younger grades require more immersive and interactive learning experiences to bridge the gap between theoretical knowledge and real-world ecological challenges. The predominance of zero and basic knowledge levels underscores the importance of implementing tailored educational strategies to enhance students’ comprehension and foster long-term conservation values.

#### 3.4.2 Knowledge levels of 3rd and 4th grade students (post-education program)

The survey revealed intermediate knowledge as the most common level among 3rd and 4th-grade students (6 out of 15, *n* = 15), with only 2 students demonstrating advanced understanding. Basic knowledge was observed in 4 students, while 3 students lacked any knowledge about key topics such as “What is a wetland?” and “Flora and fauna of the wetland.” These findings highlight the need for immersive, hands-on learning experiences, which have been shown to improve ecological awareness and understanding in young learners (Damerell et al., 2013; Sukhontapatipak and Srikosamatara, 2012).

**Figure 7:**
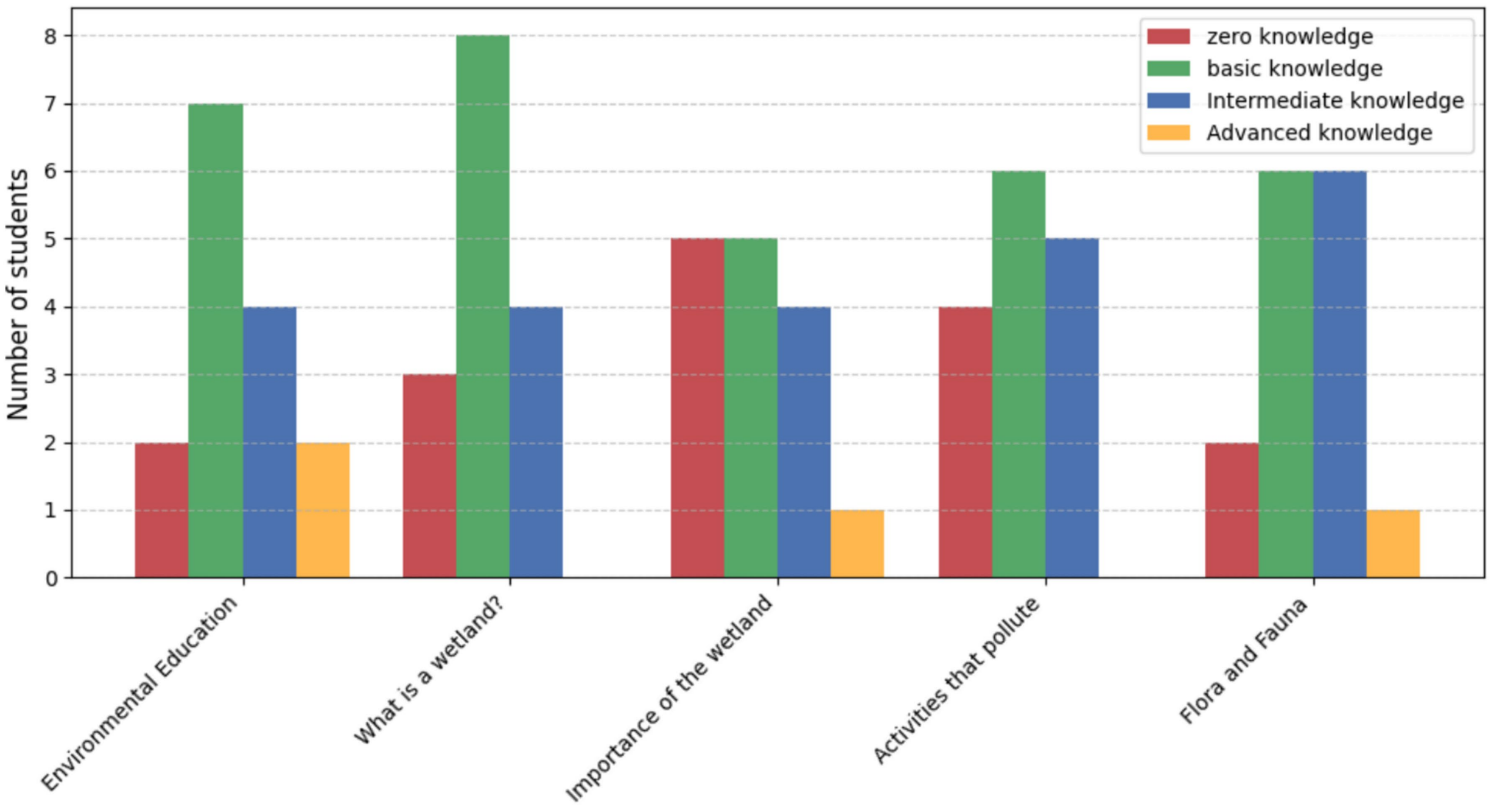
Knowledge levels of 3rd and 4th-grade students (*n* = 15) after the educational program on environmental conservation.

Students showed limited awareness of pollution impacts, with only 1 student displaying advanced knowledge (Figure 7). These findings emphasize the importance of immersive teaching methods to foster deeper ecological connections at an early age.

#### 3.4.3 Knowledge levels pre and post program (3rd and 4th grade students)

**Figure 8:**
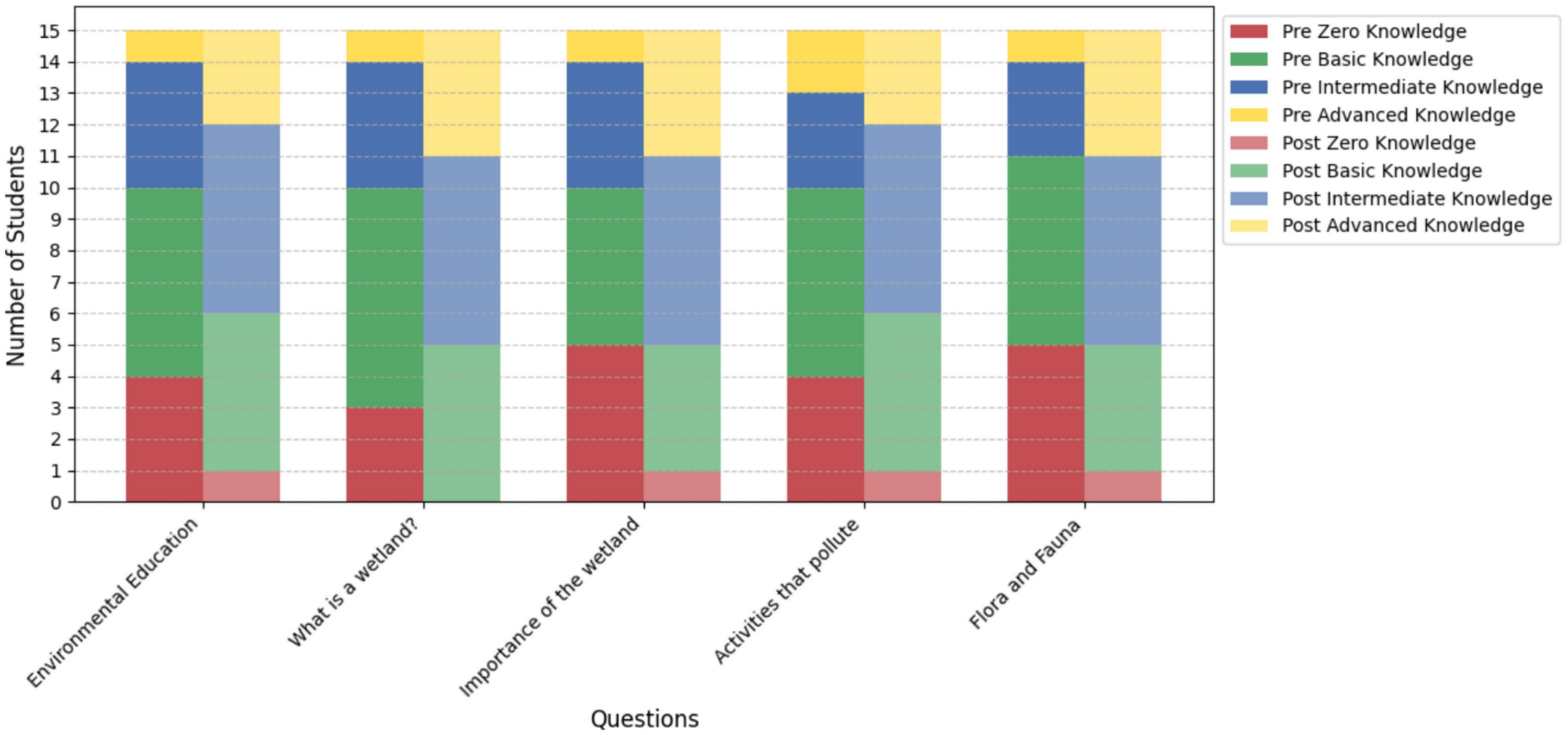
Comparison of knowledge levels in 3rd and 4th-grade students (*n* = 15) before and after the educational program.

Figure 8 illustrates the comparison of knowledge levels among 3rd and 4th-grade students before and after the implementation of the educational program. The results indicate significant progress in learning, as evidenced by a substantial decrease in the number of students classified as having “Zero Knowledge.” For instance, in the question “*What is a wetland?*”, the number of students in this category dropped from 3 before the program to 0 after its implementation.

At the same time, there is a notable increase in the “Intermediate Knowledge” and “Advanced Knowledge” levels. In the question *“Flora and Fauna,”* the number of students with advanced knowledge rose from 1 to 4, reflecting a deeper understanding of the evaluated topics. Similarly, students with intermediate knowledge showed consistent improvement across most questions, indicating strengthened comprehension of key program concepts.

The comparison between pre- and post-program results highlights a clear transition from lower knowledge levels (Zero and Basic Knowledge) to higher levels (Intermediate and Advanced Knowledge). This is evident in questions such as *“Importance of the wetland”* and *“Activities that pollute,”* where the educational program succeeded in helping more students achieve higher knowledge levels.

Overall, these results underline the positive impact of the program on the environmental education of 3rd and 4th-grade students. The reduction in lower knowledge levels, combined with an increase in students achieving advanced knowledge, validates the program’s effectiveness in fostering a meaningful understanding of the evaluated topics, paving the way for long-term environmental commitment (Figure 8).

This section presents a comparison of knowledge levels within each group (*n* = 15 per group) before and after the educational program.

#### 3.4.4 Knowledge levels of 5th and 6th grade students pre-education program

**Figure 9:**
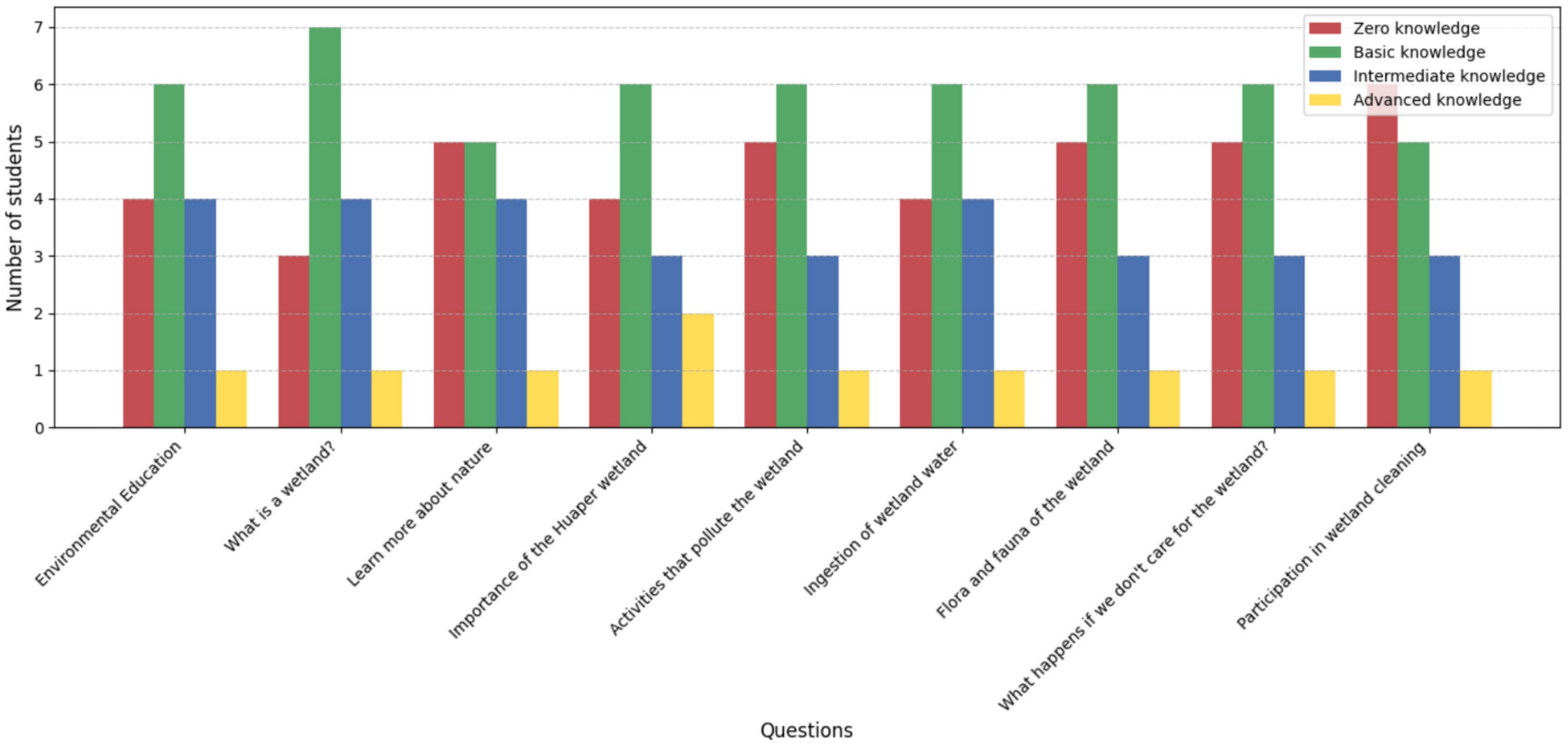
Knowledge levels of 5th and 6th-grade students (*n* = 15) prior to the educational program on environmental conservation.

The 5th and 6th-grade group (*n* = 15) demonstrated a predominantly basic and intermediate knowledge level before the implementation of the educational program. Among these 15 participants, basic knowledge was the most observed category across most topics, such as “Flora and fauna of the wetland” and “Activities that pollute the wetland.” Intermediate knowledge followed closely, with students exhibiting some understanding of “Environmental education” and “What happens if we do not care for the wetland?” However, advanced knowledge was the least common, observed in only a few students for topics like “Learn more about nature.” Notably, zero knowledge was still evident in students regarding critical topics like “Flora and fauna of the wetland” (Figure 9).

These results highlight the need for targeted educational interventions to address the lack of foundational knowledge, particularly in areas such as biodiversity and human impacts on wetlands. The low number of students with advanced knowledge underscores the potential for significant improvement through experiential learning approaches.

#### 3.4.5 Knowledge levels of 5th and 6th graders (post-education program)

**Figure 10:**
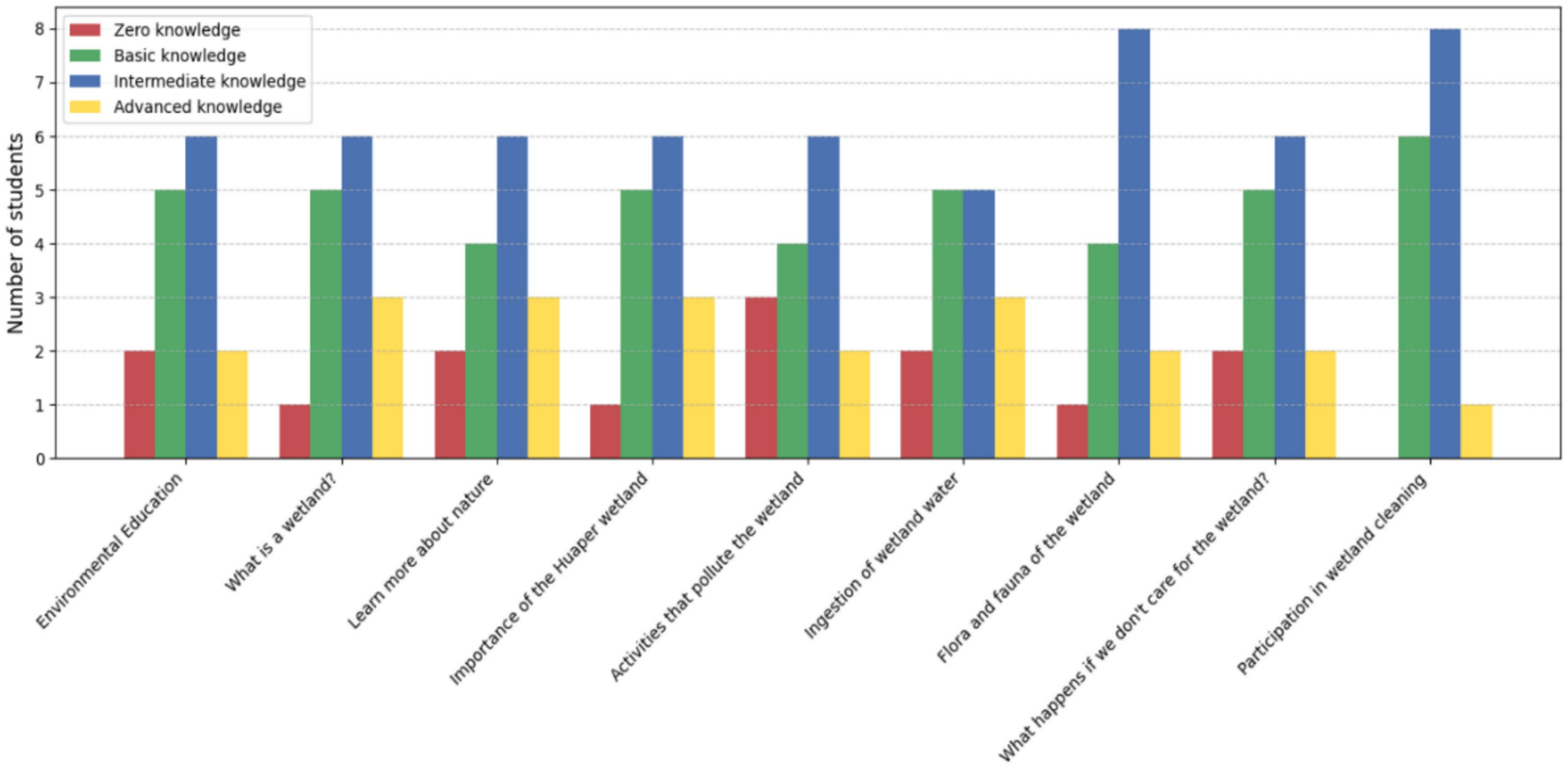
Knowledge levels of 5th and 6th-grade students (*n* = 15) after the educational program on environmental conservation.

The 5th and 6th-grade group (*n* = 15) exhibited a more balanced distribution of knowledge. Among these 15 participants, intermediate knowledge was again the most prevalent level (6 students), while 3 students demonstrated advanced understanding across topics like “The importance of the Huaper wetland” and “Pollution activities.” However, 1 student lacked knowledge entirely, indicating room for improvement even at higher grade levels (Figure 10).

These results suggest that while older students benefited from the program’s hands-on approach, additional focus on complex topics such as biodiversity conservation is needed to enhance their understanding and commitment to wetland protection (Papapanagou et al., 2005).

#### 3.4.6 Knowledge levels pre and post program 5th and 6th grade

The graph illustrates the changes in knowledge levels among 5th and 6th-grade students before and after the educational program. The data is divided into four categories: Zero Knowledge, Basic Knowledge, Intermediate Knowledge, and Advanced Knowledge, evaluated for each question. Post-program results show a clear reduction in the Zero Knowledge category across all questions. For instance, in the question “What is a wetland?” the number of students with zero knowledge decreased from 3 to 1.

The Basic Knowledge category also saw a slight reduction post-program for some questions, such as “Flora and fauna of the wetland,” indicating a progression to higher knowledge levels. The Intermediate Knowledge category exhibited the most significant growth, with increases across all questions. For example, in “Learn more about nature,” the number of students in this category rose from 6 to 8.

**Figure 11:**
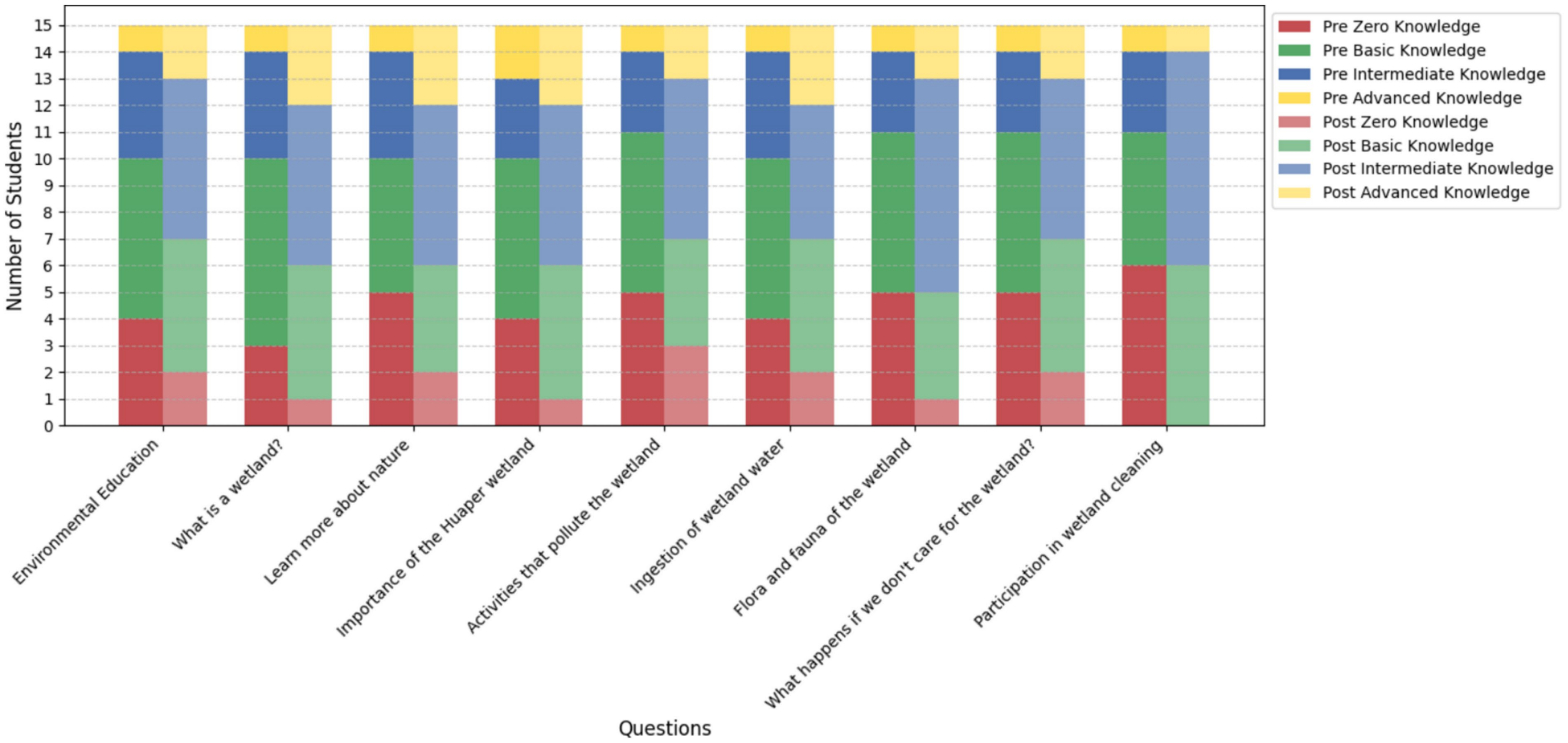
Comparison of knowledge levels in 5th and 6th-grade students (*n* = 15) before and after the educational program.

Similarly, the Advanced Knowledge category displayed noticeable growth, particularly in questions like “Importance of the Huaper wetland,” where the number of students doubled from 3 to 6. These findings further validate the program’s impact, demonstrating a clear progression from lower knowledge levels (Zero and Basic Knowledge) to higher levels (Intermediate and Advanced Knowledge) (Figure 11). The results align with the program’s aim to deepen students’ understanding of environmental issues and promote engagement in conservation efforts.

### 3.5 Integrated findings and educational impact

The combination of ecological and educational results highlights the interconnected challenges and opportunities in wetland conservation:

- Ecological Health: The physicochemical analysis underscores the wetland’s role as a buffer system but reveals stressors like low DO levels that threaten biodiversity. These findings call for immediate action to enhance water quality and protect aquatic habitats.
- Educational Gaps: Survey results indicate that students generally possess intermediate knowledge of wetland conservation but lack depth in advanced topics like pollution impacts and biodiversity. Younger students particularly struggled with these areas, emphasizing the need for age-appropriate, experiential learning strategies.
- Program Successes: Despite these gaps, the educational program succeeded in fostering interest and foundational knowledge among students. The integration of theory and practice enabled students to connect classroom concepts with real-world challenges, a key step toward long-term conservation engagement.

By addressing both ecological stressors and educational gaps, the findings support the development of integrated conservation and education strategies that engage local communities while preserving the Huaper wetland for future generations.

## 4 Discussion

The results of this study emphasize the importance of wetlands as vital ecological systems and the need for educational programs to foster conservation awareness. The implementation of the educational program at the Huaper wetland demonstrates promising outcomes as a case study in environmental education. Its alignment with constructivist principles and its potential to support the United Nations Sustainable Development Goals (SDGs) provide a framework for application in other regions.

### 4.1 Environmental education as a catalyst for conservation

Community participation and environmental education are pivotal in wetland conservation. Previous studies highlight that fostering a sense of shared responsibility between local communities and authorities enhances ecosystem stewardship (Calhoun et al., 2003; Saluja et al., 2023). The findings of this study corroborate these insights, demonstrating that the integration of hands-on learning activities with theoretical lessons significantly improved students’ understanding of wetland ecosystems.

For instance, students participating in water quality analysis and biodiversity mapping showed increased engagement and comprehension, aligning with research that emphasizes the importance of experiential learning (Truong, 2021). One student reflected, “I did not realize how important the wetland is for our water until we tested it,” underscoring the program’s impact on connecting abstract ecological concepts with tangible experiences.

Conversely, limited understanding of wetland ecosystem services among visitors highlights a persistent gap in conservation education. Addressing this gap through interpretive signage, on-site awareness campaigns, and workshops could enhance public understanding and attitudes toward conservation (Wei and He, 2018). As noted by Sinthumule (2022), improving perceptions of wetlands’ ecological and economic value is essential for long-term sustainability.

### 4.2 Challenges and opportunities in wetland management

Interviews with local authorities revealed that the management of the Huaper wetland is constrained by limited municipal involvement and inadequate infrastructure. These findings mirror global challenges faced by wetlands under anthropogenic pressures (Muema, 2019). Despite these challenges, the willingness of local stakeholders to support educational and conservation initiatives presents an opportunity for collaborative approaches.

While this study provides a valuable baseline for understanding water quality dynamics in the Huaper Wetland, the limited number of sampling sites restricts its ability to fully capture spatial heterogeneity. Future studies should consider increasing the number of sampling locations within each mesohabitat, incorporating microhabitat-level assessments, and expanding temporal coverage to account for seasonal variations. Sampling should continue until the error stabilizes around the mean, a commonly applied method to define an appropriate sample size (Magnusson and Mourão, 2004). These improvements would enhance the robustness of ecological characterizations and contribute to a more comprehensive assessment of wetland health.

Collaborative frameworks that integrate community involvement, educational institutions, and municipal support are essential for addressing these challenges (Adhya and Banerjee, 2020). Incentive-based strategies, such as Payments for Ecosystem Services (PES), could further enhance community engagement while addressing resource limitations (Maithya et al., 2021). Developing ecotourism programs could generate economic benefits while promoting conservation.

### 4.3 Alignment with SDGs and long-term impact

This study aligns with several SDGs, particularly Goal 4 (Quality Education) and Goal 15 (Life on Land). The educational program demonstrated the transformative potential of experiential learning to instill conservation values and promote sustainable practices. Field activities such as pollution clean-ups and water sampling fostered critical thinking and problem-solving skills among students, as emphasized by Ramírez and Santana (2019).

However, achieving long-term impact requires sustained efforts to monitor and evaluate the retention of knowledge and behavioral changes over time. Regular follow-up assessments, such as biannual surveys, could provide valuable insights into the effectiveness of educational interventions. Additionally, integrating supervised conservation activities within school curricula could strengthen students’ environmental literacy (Chaves, 2020).

### 4.4 Recommendations for future conservation and education strategies

The Based on the findings, the following recommendations are proposed:

- **Enhanced Community Engagement**: Expand educational initiatives to include community workshops, interpretive signage, and on-site awareness campaigns, fostering broader conservation awareness (Prochaska and Britton, 2020).
- **Ongoing Monitoring**: Establish continuous monitoring programs for water quality and wetland health, enabling adaptive management strategies.
- **Integrated Conservation Frameworks**: Develop collaborative approaches involving educational institutions, local authorities, and community members to address ecological and socioeconomic challenges.
- **Longitudinal Studies**: Conduct follow-up evaluations to assess knowledge retention and behavioral changes among participants, providing a measure of the program’s long-term effectiveness.

By addressing ecological stressors and educational gaps, this study highlights the potential for integrated strategies to preserve the Huaper wetland as a vital natural resource. These efforts not only support wetland conservation but also empower communities to take active roles in environmental stewardship, laying the foundation for sustainable futures.

## 5 Conclusion

This study underscores the ecological and educational significance of the Huaper Wetland, addressing key challenges in its conservation. The physicochemical analysis revealed ecological stressors, such as critically low dissolved oxygen levels (0.13–0.20 ppm) and elevated ion concentrations (conductivity: ~866 μS/cm), which pose risks to aquatic biodiversity and ecosystem functionality. These findings highlight the need for interventions focused on improving water quality through waste management, nutrient input control, and continuous monitoring of ecological parameters. Additionally, the stable pH (6.93) and temperature (~19–20°C) suggest a buffering effect within the wetland, contributing to its ability to sustain aquatic life despite external pressures.

However, it is important to acknowledge that the characterization of water quality was based on only two sampling locations, which limits the ability to fully capture spatial variability within the wetland. The findings should therefore be interpreted as preliminary and descriptive, rather than providing a comprehensive ecological assessment. Future studies should expand the number of sampling sites across different mesohabitats, conduct seasonal monitoring, and refine the methodology to enhance the robustness of wetland assessments.

The educational component demonstrated the transformative potential of experiential environmental education. By integrating practical activities like water sampling and biodiversity observation with classroom learning, the program facilitated significant improvements in students’ understanding of conservation concepts. This approach is consistent with constructivist learning theories, which emphasize the value of connecting theoretical knowledge with real-world applications. Beyond individual learning outcomes, the program played a pivotal role in raising community awareness, highlighting the intersection between education and collective conservation action.

The findings reinforce the importance of environmental education not only as a tool for fostering ecological stewardship but also as an effective method for enhancing learning within a constructivist framework. Experiential learning equips students with the critical thinking skills and motivation required to address environmental challenges, positioning education as a cornerstone for sustainable conservation strategies.

To achieve holistic and participatory wetland conservation, this study underscores the necessity of strengthening community engagement through education, fostering stakeholder collaboration, and addressing ecological stressors. Emphasizing the wetland’s socioeconomic importance—such as its role in agriculture, water security, and cultural heritage—ensures that conservation efforts align with local priorities, garnering community support and commitment.

Finally, this study provides a replicable model for integrating experiential environmental education into conservation strategies. By engaging students and the broader community in practical learning, it paves the way for sustainable wetland management. Moving forward, expanding educational programs, implementing incentive-based mechanisms like Payments for Ecosystem Services (PES), and establishing long-term monitoring protocols are critical for safeguarding the Huaper Wetland’s ecological integrity and resilience. This integrated approach not only supports the preservation of the wetland but also contributes to advancing environmental education as a catalyst for global sustainability goals.

## Data Availability

The raw data supporting the conclusions of this article will be made available by the authors, without undue reservation.
